# The Serum Diagnosis of Typhoid Fever

**Published:** 1898-09

**Authors:** Michael Hoke

**Affiliations:** Atlanta, Ga.


### THE SERUM DIAGNOSIS OF TYPHOID FEVER.

BY MICHAEL HOKE, M.D.,
ATLANTA, GA.

Since 1886 there have been many efforts to utilize the presence of the typhoid bacillus for the diagnosis of typhoid fever, but until the application of the test by Widal, June, 1896 (the nature of it and technique having been described by others), nothing practical for the practitioners had been developed, because the tediousness of and uncertain results from all previous methods had prevented their adoption.

The steps in the development of this test were as follows:

In 1889 Charrin and Roger noticed the clumping of the bacillus pyocyanicus when grown in the serum of an animal immune against this organism.

In 1894, Pfeiffer introduced cholera spirilla into the peritoneal cavity of a guinea-pig immune against the disease. Examination of the peritoneal fluid showed that the organisms therein soon lost their motility, agglomerated and soon broke up into fine granules. In 1896 Pfeiffer and Kolb demonstrated the phenomenon for typhoid infection also.

In 1896 Durham, and afterwards Durham and Gruber, published papers explaining the nature of the action of immune sera on bacteria outside of the animal body. The microscopic and macroscopic tests were described. It was shown that by the test it was possible to differentiate between bacterial species, and to determine whether one had had an attack of typhoid fever or cholera previously. Widal was the first to apply this developed test to the diagnosis of the disease in the early stage of infection.

He withdrew blood from a superficial vein at the elbow of the patient with a syringe, under aseptic precautions. The serum, after the blood had coagulated, was decanted and added to a bouillon culture of the typhoid bacillus in the proportion of one part serum to fifteen parts bouillon. The culture so treated was placed in a thermostat at 37° C. The serum was also added in the same proportion to a bouillon culture of the colon bacillus. This culture was placed in the thermostat with the other one. In four or five hours the tube containing the colon bacillus became cloudy, while the one containing the typhoid bacillus was clear. At the end of twenty-four hours the tube containing the colon bacillus became cloudy throughout, while the typhoid bacillus culture was still clear, the bacteria having been precipitated to the bottom as whitish flakes.

A drop of the culture of the colon bacillus examined under the microscope showed isolated bacteria, activity motile. A drop of the culture of the typhoid bacillus, examined microscopically, showed scattered clumps of bacteria which had lost their motility and were agglutinated.

These two phenomena, the formation of flakes visible to the eye in the culture and precipitated to the bottom, and the agglutination, agglomeration and immobilization as seen by the examination of a drop of the culture with the lens, constitute the characteristics of the macroscopic and microscopic tests, respectively. Sometimes there is complete clumping and partial loss of motion or complete immobility and partial clumping.

The value of this test for diagnostic purposes depends upon the specificity of the change in the blood brought about by infection with the typhoid organism or intoxication with its products.

The normal blood possesses in a certain degree the power of agglutinating the typhoid bacillus, but the result of infection is to increase this power far beyond that which has been observed for the normal blood.

What the substance is in the blood which possesses the agglutinative power is not known. Blood-serum deprived of globulin looses the power, while the globulin retains it. When the fibrin and globulin are separated, each possesses the property, while the plasma left does not.

The agglutinative property in typhoid fever has been found in blister-serum, pleural, pericardial, peritoneal, inflammatory and edematous fluids; in the milk, aqueous humor, urine and stools. The blood acquires the agglutinative property usually by the end of the first week after the entrance of the organism or its products. This power increases irregularly during the infection, gradually disappearing weeks or months or years after recovery. In some instances it occurs as early as the fourth day, in others not until the end of the third week or later, rarely not at all.

The agglutinative property is not destroyed by drying the blood for months. It resists the action of sunlight. Prolonged heating at 60° C. weakens it, and heating at 75° for ten minutes destroys it.

The blood may be obtained by pricking the tip of the finger or the lobe of the patient's ear. Several large drops are then collected on a clear slide, which is tilted so as to partially separate the clot and serum. The blood is allowed to dry. The test may be made at once or at leisure. Another way is to withdraw the blood from a vein of the arm with a syringe under aseptic precautions. The blood is forced out into a tube; the serum separates from the clot. This method gives an abundance of serum. Still another method is to collect the blood from the pricked finger in a small capillary tube. The serum separates and may be dropped in quantity desired.

The use of the dried specimen has given excellent results, and possesses the advantage of ease of collection, freedom from contamination, and readiness of transportation. For precision, it possesses the disadvantage of inaccuracy in the quantitative dilutions, but for all practical purposes it suffices.

If the dried specimen is used, it is brought into solution by mixing it with about five times the quantity of water; then a drop of this mixture is placed on a cover-glass, and to it is added a drop of bouillon culture of the typhoid bacillus eighteen hours old, or a drop of a suspension in bouillon of an agar culture of the same age. This cover-glass is inverted over a hollow slide and examined with a one-half oil of immersion lens.

If one uses the capillary tube or syringe in obtaining the specimen, absolute accuracy in the quantitative dilutions becomes possible. If a large quantity of serum has been obtained, it is sucked up with a graduated pipette to the point marked 1, and then blown out into a watch-glass. From a bouillon culture of a typhoid bacillus is sucked up as many times the quantity of culture or serum as is desired, the number depending upon the dilution wished. This is mixed with the serum in the watch-glass, and a drop or two of the mixture is used to make the examination.

If the capillary tube is used, a drop of the serum from it is added to just as many drops of the culture as will give the desired dilution. The capillary tube used in dropping the culture must be of the same diameter as the one used in collecting and dropping the serum. A cover-glass is prepared as above and examined.

If the reaction develops slowly, one soon notices that many of the bacilli are not as actively motile as they should be. Soon they are gathered here and there in groups of twos, threes, fours, etc.; the bacilli stick to one another, tugging to get loose. The paralyzing of motility and clumping is progressive. Finally, what was at first a field of isolated, actively-motile bacteria becomes one in which organisms are gathered here and there in clumps, with clear spaces between, and partially or completely devoid of motility. If the reaction has been complete and immediate, as soon as the slide is examined the organisms are seen to be paralyzed and stuck together in clumps. The various degrees between the immediate and delayed reaction are observed according to the quantity of the agglutinative substances in the blood and the degree of dilution.

The normal blood-serum possesses the agglutinative power, if diluted in proportion of not more than one part serum to ten parts culture. On account of this fact it is necessary to make the dilution sufficiently great to preclude the possibility of an error from this source. Various standards of dilution have been suggested, from one to twenty-five to seven to fifty. In only a few cases of typhoid fever has the reaction been observed in dilutions less than one to fifty. Very few cases would be missed. A negative result in a preliminary test with equal parts serum and culture excludes typhoid fever. If the examination be positive and immediate with dilutions one to fifteen or one to twenty, the diagnosis of typhoid fever can be made; but, to be absolutely certain, other tests should be made with higher dilutions if the quantity of serum suffices. If only one test is made, it should be with a dilution of one to fifty with a time limit of one to two hours.

In a total of 422 cases of typhoid or suspected typhoid fever collected by the New York health department, reported by fifteen observers, 404 were positively diagnosticated to be typhoid fever or not—312 positively, 92 negatively; or, in about 95 per cent. the diagnosis was confirmed by the test on the first examination.

The ease with which typhoid fever may deviate from the classical type and the existence of a great number of symptoms, designated by the term "typhoid," which may be present in profound infections other than typhoid bacillus, are but evidences of the confusion which may sometimes be in the mind of even the most skilled practitioner over the entity of a doubtful febrile attack. The similarity between certain cases of meningeal, peritoneal, and acute miliary tuberculosis, ulcerative endocarditis, estivo-autumnal type of malaria, septic infections, etc., are well known. The value of this test in clearing up the doubt in these cases has been tnoroughly demonstrated in the municipal laboratories and the hospitals of the large cities in this country and abroad for the last two years. Particularly should it be of value in the South for elucidating the nature of many doubtful short fevers.

This incomplete review and description of the technique of this test may be pardoned if it point out the tardiness of its adoption.

*267 Peachtree St.*

